# Functional connectivity in ADHD children doing Go/No-Go tasks: An fMRI systematic review and meta-analysis

**DOI:** 10.1515/tnsci-2022-0299

**Published:** 2023-12-31

**Authors:** Sihyong J. Kim, Onur Tanglay, Elizabeth H. N. Chong, Isabella M. Young, Rannulu D. Fonseka, Hugh Taylor, Peter Nicholas, Stephane Doyen, Michael E. Sughrue

**Affiliations:** Centre for Minimally Invasive Neurosurgery, Prince of Wales Private Hospital, Sydney, Australia; Omniscient Neurotechnology, Sydney, Australia; National University of Singapore Yong Loo Lin School of Medicine, Singapore, Singapore

**Keywords:** ADHD, Go/No-Go, connectivity, networks, ALE, meta-analysis, fMRI

## Abstract

Attention deficit hyperactivity disorder (ADHD) is one of the most common neurodevelopmental disorders diagnosed in childhood. Two common features of ADHD are impaired behavioural inhibition and sustained attention. The Go/No-Go experimental paradigm with concurrent functional magnetic resonance imaging (fMRI) scanning has previously revealed important neurobiological correlates of ADHD such as the supplementary motor area and the prefrontal cortex. The coordinate-based meta-analysis combined with quantitative techniques, such as activation likelihood estimate (ALE) generation, provides an unbiased and objective method of summarising these data to understand the brain network architecture and connectivity in ADHD children. Go/No-Go task-based fMRI studies involving children and adolescent subjects were selected. Coordinates indicating foci of activation were collected to generate ALEs using threshold values (voxel-level: *p* < 0.001; cluster-level: *p* < 0.05). ALEs were matched to one of seven canonical brain networks based on the cortical parcellation scheme derived from the Human Connectome Project. Fourteen studies involving 457 children met the eligibility criteria. No significant convergence of Go/No-Go related brain activation was found for ADHD groups. Three significant ALE clusters were detected for brain activation relating to controls or ADHD < controls. Significant clusters were related to specific areas of the default mode network (DMN). Network-based analysis revealed less extensive DMN, dorsal attention network, and limbic network activation in ADHD children compared to controls. The presence of significant ALE clusters may be due to reduced homogeneity in the selected sample demographic and experimental paradigm. Further investigations regarding hemispheric asymmetry in ADHD subjects would be beneficial.

## 1 Significance statement

Attention deficit hyperactivity disorder (ADHD) is one of the most common neurodevelopmental disorders diagnosed in childhood, with key features of impaired behavioural inhibition and sustained attention. However, there has been no neuroanatomical specificity regarding the localisation of motor inhibition; inhibitory deficits have been attributed to generalised brain areas and even entire lobes. This article quantitatively consolidates effects across various Go/No-Go experiments into holistic, network-based nomenclature to be easily understood by clinicians in the field, with an aim to translate these outcomes in the field of paediatrics, psychiatry, and neurology. We used a coordinate-based meta-analysis that specifically focuses on motor inhibitory coordinates during a Go-/No-Go task in the ADHD paediatric population, ensuring an objective, statistically based approach in an effort to reduce heterogenicity seen in previous task-based functional magnetic resonance imaging (fMRI) studies. Through this approach, we aimed to enhance clinical understanding of the neural correlates of ADHD.

## 2 Introduction

ADHD is a prevalent neuropsychiatric condition that is frequently diagnosed in childhood. It is characterized by a persistent pattern of age-inappropriate behaviour such as inattention, impulsivity, and hyperactivity, leaving profound impacts on development. Since its first recognition in the DSM classification in the 1980s, significant work in the fields of behavioural science and neuropsychiatry advanced and refined the understanding of the ADHD phenotype. In particular, the use of fMRI during cognitively demanding tasks such as the Go/No-Go paradigm has shed light on the neural correlates of certain functions, which may be impaired in patients with ADHD relative to controls. Indeed, various fMRI investigations have attributed deficits in motor inhibition and selective attention during the Go/No-Go task to decreased activation in the anterior and posterior cingulate, large portions of the frontal cortex, the supplementary motor area (SMA), the dorsolateral prefrontal cortex (DLPFC), and the basal ganglia to list a few anatomical areas [1,2,3,4,5,6,7,8]

However, these earlier task-based fMRI investigations can often yield inconsistent results, perhaps due to minor differences in the performance of the task and the sample demographic. Furthermore, recent meta-analyses that seek to summarise the myriad of task-based fMRI data in the literature report a significant degree of heterogeneity, attributing the core neurological deficits in ADHD to an expansive list of regions spanning various cortical and subcortical areas [9,10,11,12,13]. Some have suggested that this inconsistency is due to the inherent differences between neural mechanisms underlying children and adults with ADHD who are often grouped in meta-analyses [13,14,15]. Others have suggested that there is an increasing need to deviate away from abnormalities in discrete anatomical brain regions and instead shift the focus onto altered or aberrant brain connectivity between- and within-functional networks or neural circuitry [14,16,17]. This latter point is further supported by recent advances in brain mapping and connectomics that divide the brain into microscopic parcellations or nodes of interest, thus providing greater nuances and specificity to the neural correlates of ADHD beyond the level of macroscopic lobes or systems [18,19].

Considering the above, there is a need for an objective, statistically based approach to quantitatively synthesize relevant findings from the ADHD neuroimaging literature. While there have been a few meta-analyses for both task-based and resting-state fMRI for patients with ADHD, none has focused specifically on the motor inhibition correlates of children performing the Go/No-Go task. Here, we present a coordinate-based meta-analytical approach that generates activation likelihood estimates (ALEs) to ascertain probabilistic regions of interest that suggest a convergence of multiple studies finding hyper- or hypoactivation in the brain. This voxel-wise approach offers increased spatial resolution, where the foci of each ALE represent the area of the highest likelihood of activation as the centre of a three-dimensional Gaussian probability distribution [20,21]. We also labelled each focus according to Glasser’s atlas [18], a cortical parcellation scheme derived from the Human Connectome Project to provide a clinically relevant nomenclature and a network-based understanding of the neural architecture that may be involved in motor inhibition deficits in children with ADHD. Furthermore, we hypothesize that the reduced heterogeneity in this meta-analysis created by focusing specifically on children and the Go/No-Go task will yield significant results that would be relevant for the understanding of children with ADHD in the critical period of development.

## 3 Methods

### 3.1 Experimental design

This review was conducted according to the PRISMA 2020 Guidelines [22]. The review and the protocol were not registered. A preliminary search was made using the BrainMap Sleuth 3.0.4 functional imaging database in May 2023 matching the following criteria: “Imaging Modality = fMRI” and “Subject Diagnosis = ADHD.” Sleuth is software that algorithmically screens the BrainMap database for functional and structural neuroimaging results presented in 3-D stereotactic (*x*, *y*, *z*) coordinates which were appropriate for our purpose of conducting a coordinate-based meta-analysis [23,24]. Furthermore, through Sleuth’s archives, we were able to select particular studies which employed the Go/No-Go paradigm and our desired experimental contrasts of No-Go > Go which would provide fMRI foci of interests that correspond to the neural correlates of response inhibition. To cover the literature gap, an additional search in PubMed was conducted, from January 2000 up to May 2023, using the following search terms: “ADHD” AND “fMRI” AND “Go/No-Go.” Two authors independently screened each record. Studies were reviewed and included if they fulfilled the following search criteria: (1) peer-reviewed publication; (2) Go/No-Go paradigm-based fMRI study; (3) regions of interest presented as stereotactic coordinates in either Talairach or Montreal Neuroimaging Institute (MNI) coordinate space; (4) experimental contrasts must be No-Go > Go; (5) includes at least one healthy human control cohort for comparison. Studies were excluded according to the following criteria: (1) subjects’ age > 18; (2) studies tested the action of a variety of neuropsychiatric therapy, e.g. methylphenidate, atomoxetine, behavioural focus intervention, etc., with no baseline measurements; and (3) studies compared between ADHD subjects and subjects with other neurological or psychiatric diagnoses, e.g. autism spectrum disorder (ASD) or major depressive disorder. Only studies in English were included.

While formal bias assessment was not performed, given the strict inclusion and exclusion criteria used to ensure that the same methodology was used in each study, the risk of bias was low.

### 3.2 Statistical analysis

Coordinates provided in Talairach or MNI space indicating foci of strong convergence in No-Go tasks compared to Go tasks were collected for both ADHD and control groups. All data collection was performed by one author. These coordinates were categorized into four mutually exclusive groups (ADHD activation; controls activation; ADHD < controls; ADHD > controls) and then used to generate ALEs using GingerALE version 3.0.1 (http://www.brainmap.org/ale/) to analyse the probabilistic differences in brain network activation between ADHD and control subjects [20,21,25]. Following the recommended guidelines for coordinate-based neuroimaging studies [26], we performed a single-study analysis using cluster-level inference in MNI coordinate space (cluster-level: *p* < 0.05; vowel-level: *p* < 0.001; threshold permutation = 1,000) [20]. Furthermore, all Talairach coordinates were converted into MNI coordinate space before ALE generation using icbm2tal transform SPM conversion in GingerALE. We utilized the Multi-image Analysis GUI (Mango) 4.0.1 (ric.uthscsa.edu/mango) to overlay the ALE foci over an MNI-normalised brain image. To label each focus according to the cortical parcellation scheme derived from the Human Connectome Project18, a sphere was placed at the MNI coordinate of the centroid of each ALE cluster with a radius defined as 15 mm. The sphere was projected onto the HCP-MMP parcellation schema, which is also in MNI coordinates. The degree to which any local HCP parcellations fell within the ALE cluster was calculated as a percentage (percentage of the parcellation that falls within the ALE cluster). The parcellation that had the highest percentage of volume within the ALE cluster was designated as the equivalent HCP parcellation to that ALE cluster. As a result, we were able to map a region of interest suggesting hyper or hypoactivation during the Go/No-Go experiment to one of 360 subdivisions in the cortical architecture, providing specificity beyond the level of lobes and even sub-lobes. We then categorized each parcellation according to its core affiliate network based on Yeo et al.’s 7-network model of the human cerebral cortex [19]. That is, each parcellation was labelled as one of the following core networks: default mode network (DMN); central executive network (CEN); salience; dorsal attention network (DAN); limbic; sensorimotor and visual networks. “Independent group analyses” were conducted by examining the network affiliations of parcellations generated from studies that reported ADHD or control activation only. Similarly, “between-group analysis” was conducted by examining parcellations from studies that reported a comparison of ADHD and control subjects, i.e. “ADHD < controls” or “ADHD > controls.”

## 4 Results

**Figure 1:**
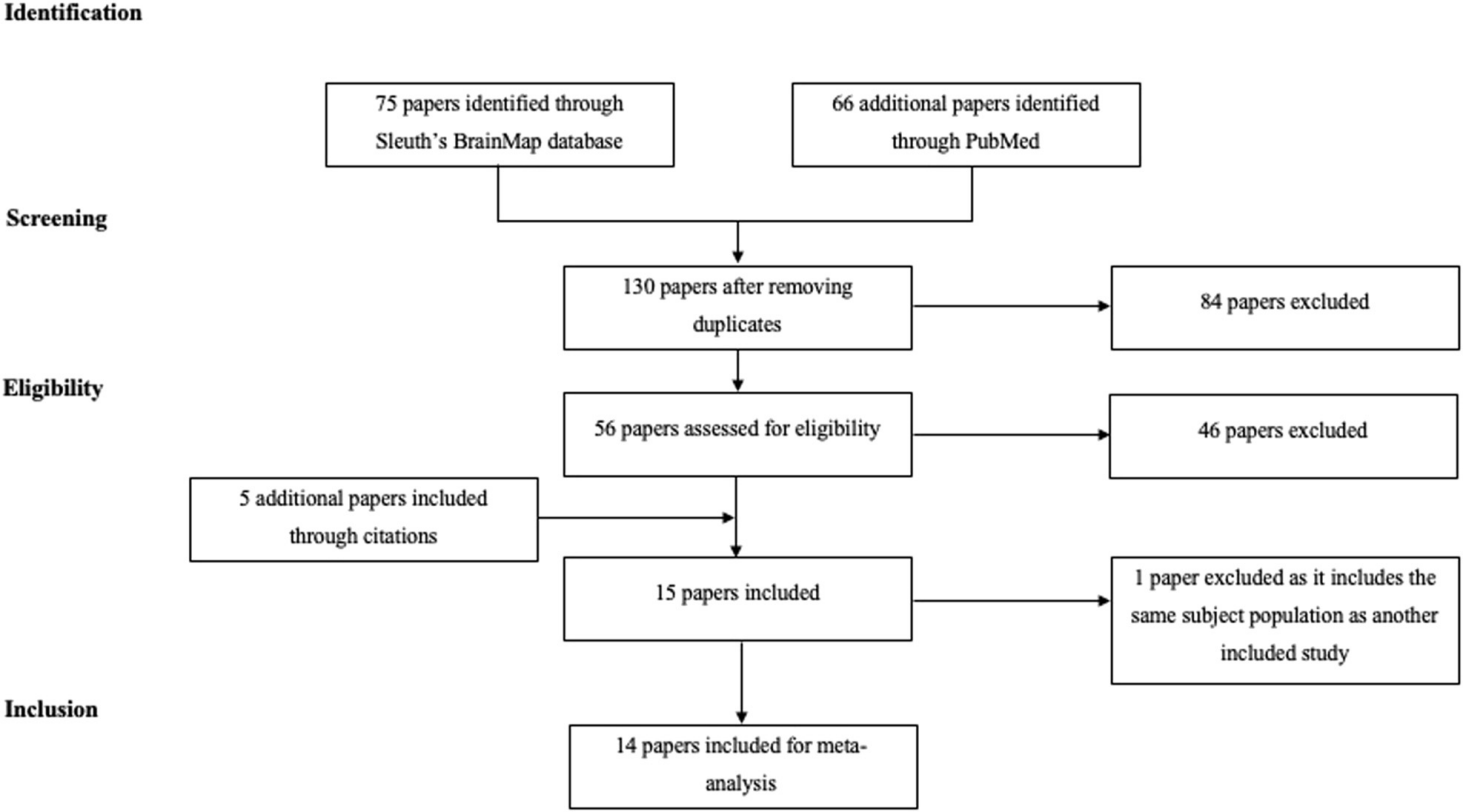
Search strategy for the meta-analytical process.

Figure 1 presents a visual representation of the study retrieval process. From the total studies which have been algorithmically determined by Sleuth’s BrainMap database to include stereotactic coordinate results of Go/No-Go experiments, 14 eligible studies met our inclusion criteria [1,3,4,5,6,7,8,27,28,29,30,31,32,33]. Some studies were excluded due to a lack of control cohorts, comparison between children with ADHD and ASD [34], or testing of neuropsychiatric therapies such as methylphenidate. One study was excluded as it included the same subject population as another study already included in our study [35]. A summary of the main findings in terms of increased brain activation in ADHD and control subjects is given in Table 1. Moreover, the characteristics of each study and the extracted MNI coordinates can be found in Supplementary Table 1. In total, there were 457 included participants (ADHD = 224; controls = 232).

**Table 1:** Main findings of increased activation in ADHD and control subjects for the 14 studies included in this meta-analysis

Study	Key findings	
	↑Activation for ADHD	↑Activation for controls
Durston et al. [4]	R superior frontal gyrus	L caudate
	R middle frontal gyrus	
	R inferior parietal lobe	
	R/L posterior cingulate	
	R/L precuneus	
	R superior temporal gyrus	
	R/L occipital cortex	
Schulz et al. [27]	R/L middle frontal gyrus	R precentral gyrus
	R/L inferior frontal gyrus	R inferior temporal gyrus
	L anterior cingulate	L superior temporal gyrus
	R/L inferior posterior lobule	R fusiform gyrus
	R precuneus	L hippocampus
		R lingual gyrus
		R/L cerebellum
Tamm et al. [7]	R/L inferior frontal gyrus	R superior frontal gyrus
	R/L frontal operculum	R middle frontal gyrus
	R/L superior temporal gyrus	R inferior frontal gyrus
	R/L middle temporal gyrus	R anterior cingulate
	R/L inferior temporal gyrus	R SMA
	L angular temporal gyrus	R angular gyrus
	L anterior cingulate	R supramarginal gyrus
Booth et al. [1]	R posterior cingulate	R superior frontal gyrus
	R superior parietal lobule	R middle frontal gyrus
	L superior temporal gyrus	R/L inferior frontal gyrus
	L middle temporal gyrus	R/L precentral gyrus
	L inferior temporal gyrus	L caudate
		L insula
		L posterior cingulate
		L fusiform gyrus
		R amygdala
Rubia et al. [8]	R superior temporal lobe	R/L infero-orbital prefrontal cortex
	L medial temporal lobe	R/L mesial prefrontal cortex
	L anterior cingulate	R/L middle frontal gyrus
	L posterior cingulate	R precentral cortex
		R medial temporal lobe
		R/L parietal lobe
		R caudate
		L cerebellum
Vaidya et al. [28]	R superior temporal gyrus	R premotor gyrus
	R insula	R/L caudate
Durston et al. [3]	R middle frontal gyrus	R superior frontal gyrus
	R IPL	R/L middle frontal gyrus
		R/L inferior frontal gyrus
		R/L anterior cingulate
		L premotor cortex
		L IPL
Pliszka et al. [5]	R/L insula	R inferior frontal gyrus
	R ventrolateral prefrontal cortex	R superior temporal gyrus
	R superior temporal gyrus	R anterior cingulate
	R/L occipital lobe	R/L ventrolateral prefrontal cortex
	R inferior parietal lobe	R posterior parietal lobe
		R/L precentral gyrus
Suskauer et al. [29]	R medial frontal wall	R medial frontal wall
		R/L occipital lobe
		R DLPFC
		R temporal–parietal junction
		R cerebellum
		R putamen
		L precentral gyrus
		R fusiform gyrus
		R anterior cingulate
		L posterior cingulate
		L precuneus
Spinelli et al. [30]	R superior frontal gyrus	R middle frontal gyrus
	R middle frontal gyrus	R superior occipital gyrus
	L inferior frontal gyrus	R angular gyrus
	L caudate	R middle temporal gyrus
	L amygdala	R parahippocampal gyrus
	L cerebellum	R hippocampus
		L precuneus
		R posterior cingulate
Ma et al. [31]	R inferior temporal gyrus	R middle frontal gyrus
	R midbrain	R inferior frontal gyrus
	R precentral gyrus	R IPL
	R postcentral gyrus	R SMA
	R calcarine	
	R/L middle occipital cortex	
	R/L inferior occipital cortex	
	R hippocampus	
Wang et al. [32]	L middle frontal gyrus	L anterior cingulate
	L middle temporal gyrus	R precentral gyrus
	L middle occipital gyrus	L middle temporal gyrus
	L putamen	R/L parahippocampus gyrus
	L posterior cingulate	
	L precuneus	
	R/L angular gyrus	
	R cerebellum	
Hart et al. [33]	L cerebellum	R/L ventrolateral prefrontal cortex
	L posterior cingulate	R/L superior temporal lobe
		R/L middle temporal lobe
		L inferior temporal lobe
		L IPL
		R/L posterior cingulate
		R/L precuneus
		R/L basal ganglia
Van Rooij et al. [6]		L superior frontal gyrus
		L inferior frontal gyrus
		L supramarginal gyrus
		R post-central gyrus
		R/L temporal-parietal junction
		R/L anterior cingulate
		L supramarginal gyrus

**Figure 2:**
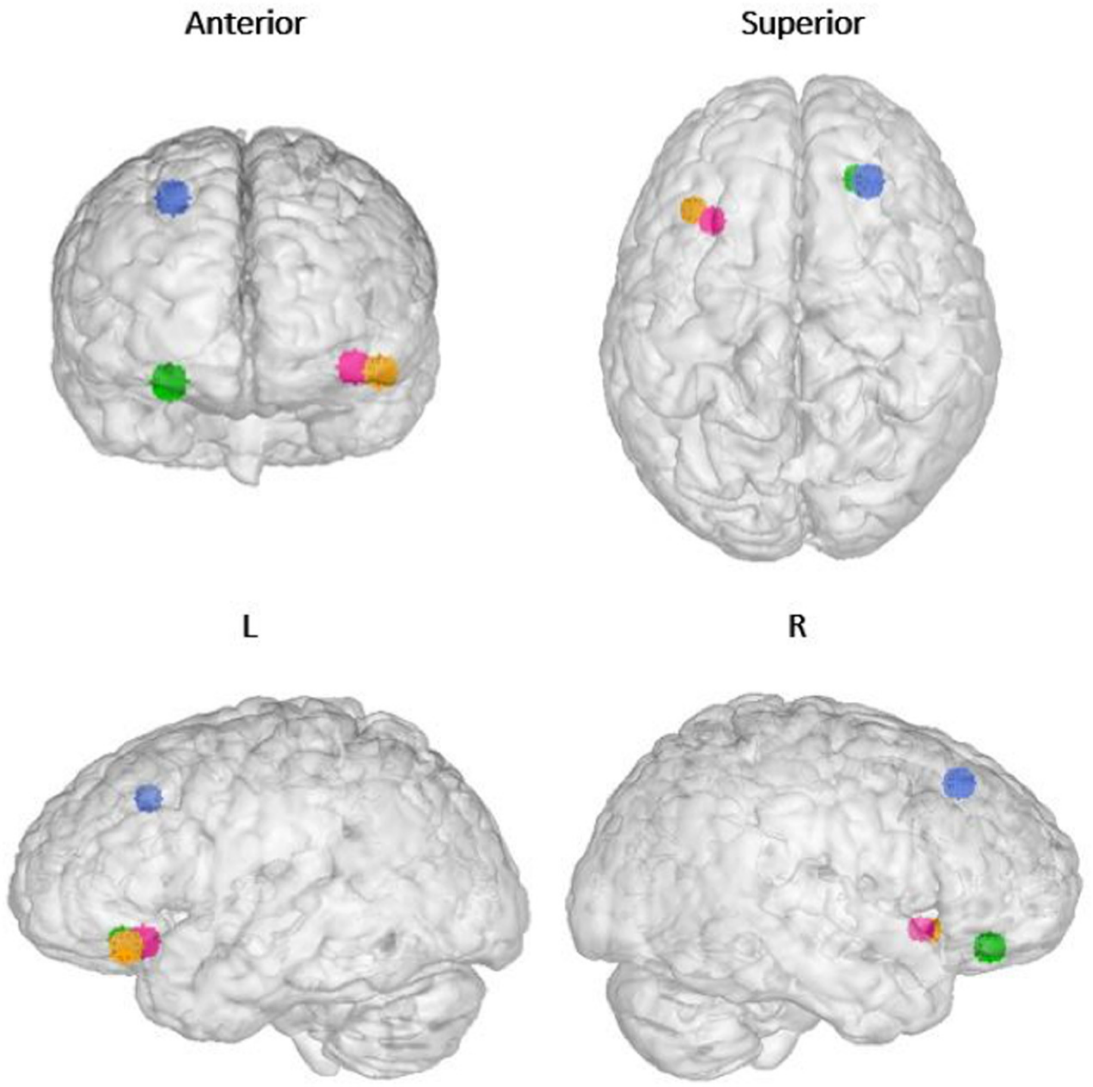
Significant clusters for “control activations” and “ADHD < controls” groups represented as voxels of 5 mm^3^ on an MNI normalised volumetric brain surface. Blue = Area 47l; Green = Area 8Av; Yellow = Area 47m; Pink = Area 47s. Area 47 and its subdivisions form part of inferior frontal gyrus and are often known for its connectivity to Broca’s area. Area 8Av (8A ventral) is located in the posterior part of the middle frontal gyrus and displays functional importance in spatial attention and interpreting visual information.

**Figure 3:**
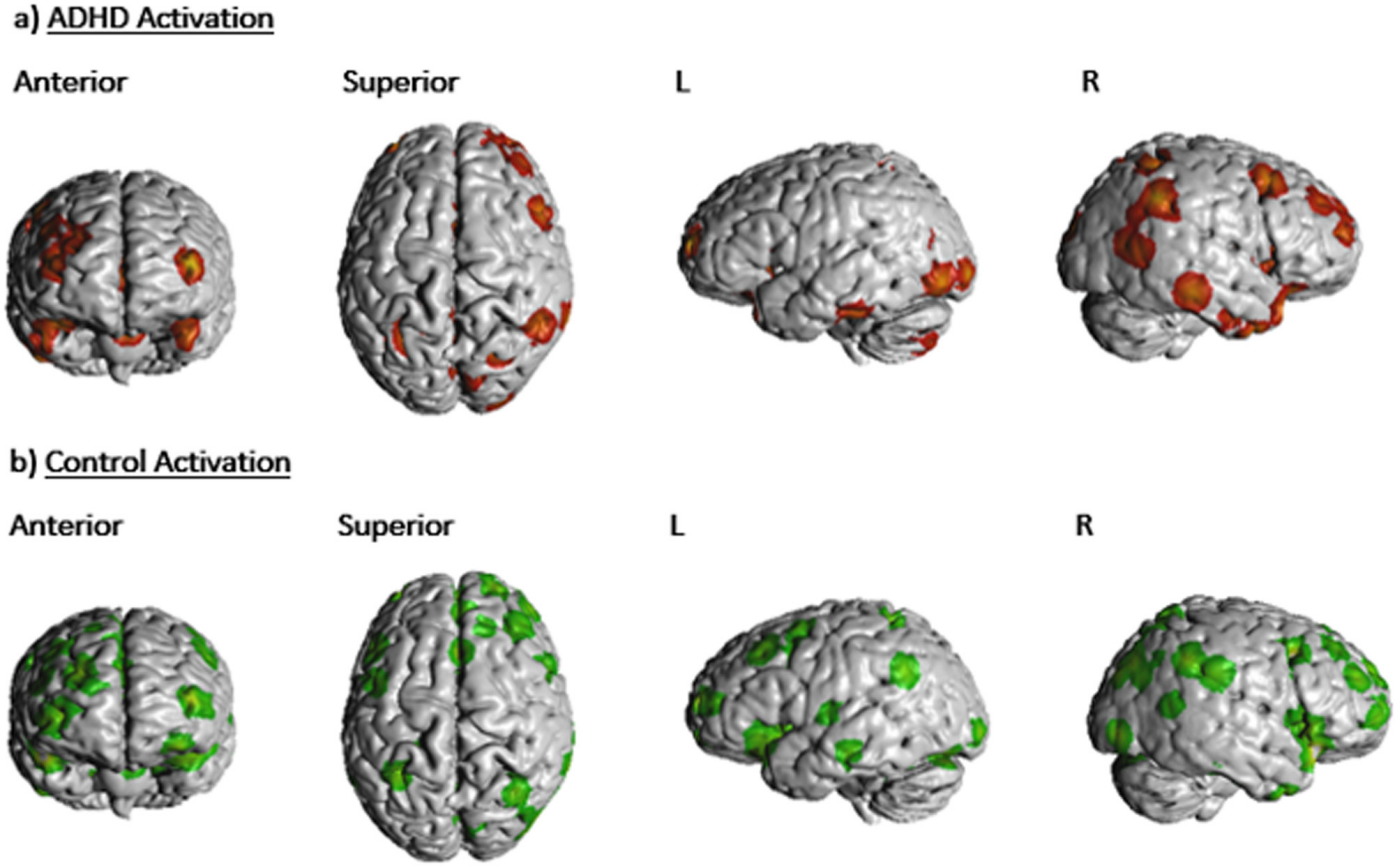
(a) Unthresholded ALE foci for “ADHD activation” group depicted on an MNI normalised brain surface. (b) Unthresholded ALE foci for “control activation” group depicted on an MNI normalised brain surface.

**Figure 4:**
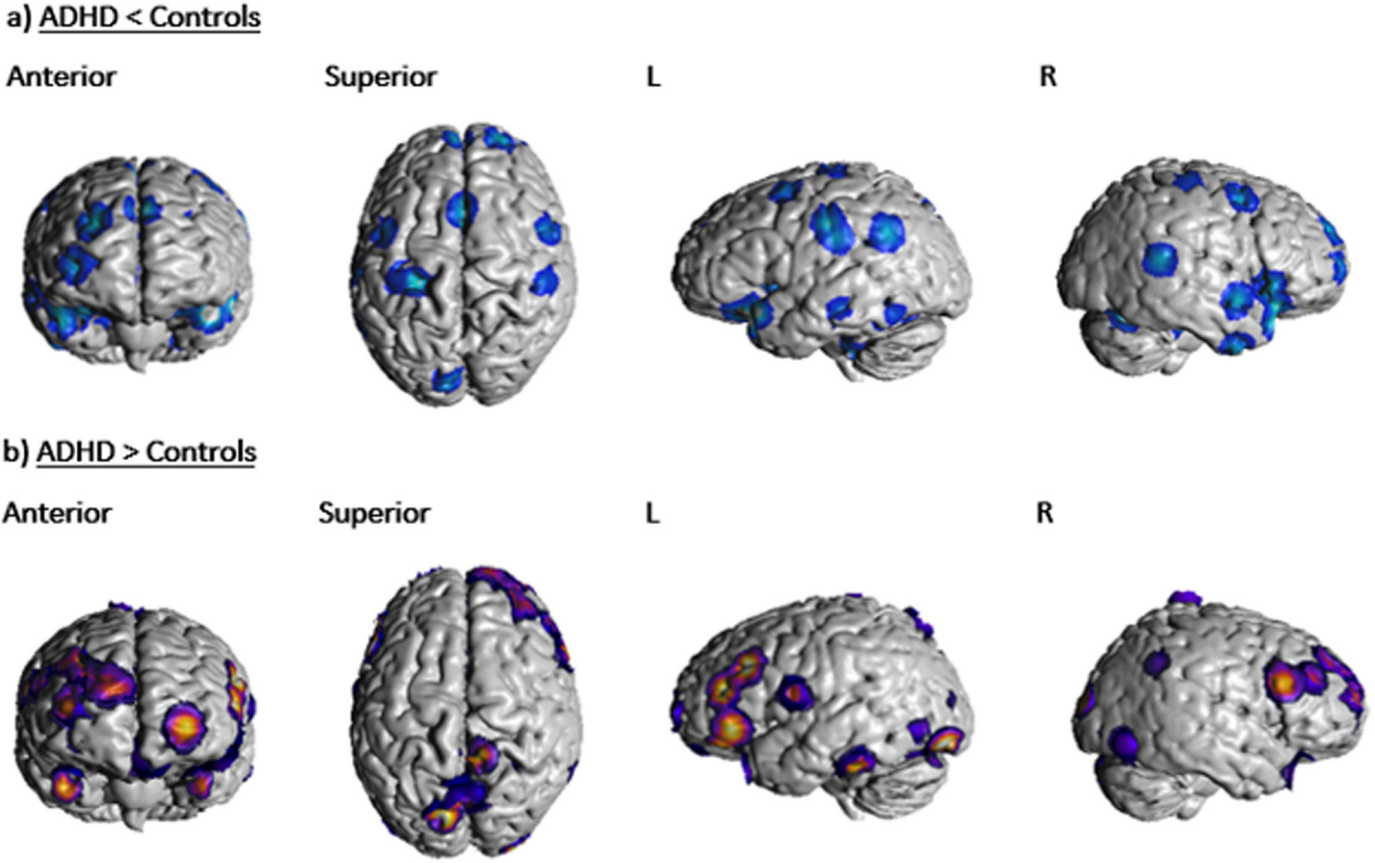
(a) Unthresholded ALE foci for “ADHD < controls” group depicted on an MNI normalised brain surface. (b) Unthresholded ALE foci for “ADHD > controls” group depicted on an MNI normalised brain surface.

**Figure 5:**
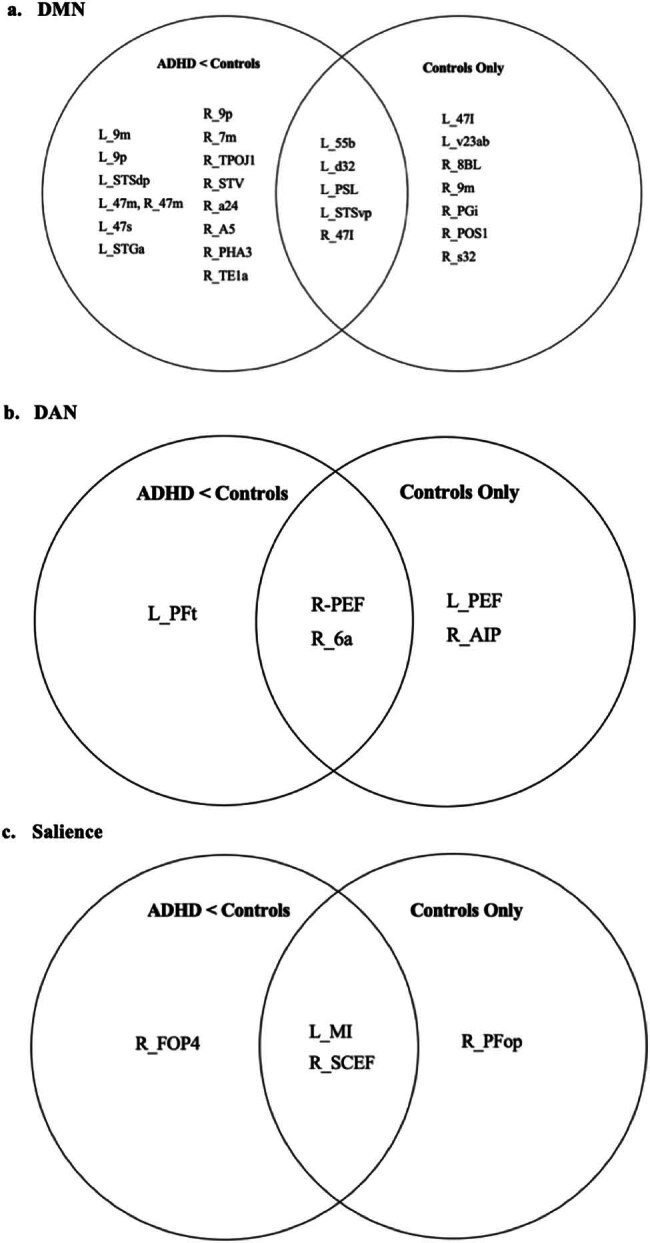
Summary of parcellations activated in the “controls activation” and “ADHD < controls” group. The union of the two groups in this Venn diagrammatic figure represents “concordant” parcellations that have been activated in both independent and between-group analyses: (a) DMN, (b) DAN, and (c) salience network.

**Figure 6:**
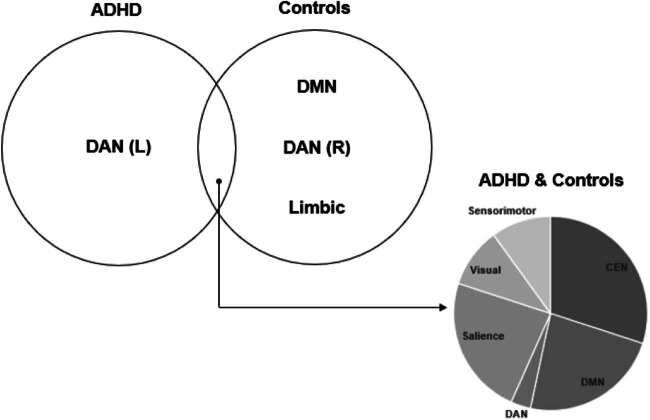
Summary of networks activated in ADHD and control subjects during the Go/No-Go paradigm.

In terms of the ALE analysis, there were no significant clusters found at the thresholding value of 0.05 and a minimum cluster volume of 528 mm^3^ for both the “ADHD activation” and “ADHD > controls” groups. Two significant clusters, both located in the right cerebrum, were detected for the “control activation” group, receiving contributions from 9 foci [3,5,6,8,28]. Cluster 1 was centred at (*x*, *y*, *z* = 39.9, 21.7, −12.7) in MNI stereotactic space with a cluster volume of 976 mm^3^ and showed involvement from the inferior frontal gyrus (50%), insula (30%), and the claustrum (17.5%). Cluster 2 was centred at (*x*, *y*, *z* = 42.5, 9.5, 35.6) with a volume of 864 mm^3^ and showed involvement from the precentral gyrus (73.9%), middle frontal gyrus (21.7%), and the inferior frontal gyrus (4.3%). One significant cluster was detected for the “ADHD < controls” group, receiving contributions from three foci [3,6,33]. This cluster corresponded to the left inferior frontal gyrus, centred at (*x*, *y*, *z* = −37.4, 22.3, −15.5) with a volume of 584 mm [3]. Parcellation matching of the above clusters to Glasser’s atlas [18] correlated with area 47 l (*x*, *y*, *z* = 42, 23, −13) and 8Av (*x*, *y*, *z* = 44, 11, 38) in the right frontal lobe and area 47 m (*x*, *y*, *z* = −37, 29, −14) and 47 s (*x*, *y*, *z* = −37, 21, −14) in the left frontal lobe. These four regions of interest are marked in an MNI-normalized volumetric brain surface in Figure 2. Furthermore, un-thresholded ALEs mapped onto an MNI-normalized brain surface for each of the four groups (ADHD activation; controls activation; ADHD < controls; ADHD > controls) are shown in Figures 3–6, respectively.

Table 2 presents the matched Glasser’s parcellations and the affiliated brain network of each activated brain foci in the “ADHD activation” and “controls activation” groups. A network-level analysis shows no significant activation of the limbic network in children with ADHD and less extensive activation of the CEN compared to controls. Furthermore, children with ADHD predominately activated left-sided parcellations of DAN, while control subjects activated predominantly right-sided parcellations. Furthermore, control subjects demonstrated more extensive activation of the DMN. Parcellation p24 of the CEN network demonstrated unique asymmetry, activated on the left for ADHD and on the right for controls. Similar findings can be applied to area POS1 of the DMN. Additionally, the left LIPv of the DAN was activated for ADHD subjects while the right LIPv of the DAN was activated for both ADHD and control subjects. A similar pattern of lateralization can be applied to area FOP4 of the salience network.

**Table 2:** List of parcellations and matched networks activated in “ADHD” and “Control” groups

	ADHD only		Control only		Both ADHD and controls	
	R	L	R	L	R	L
CEN	PFm	p24	p24	33pr	AAIC	AAIC
		31a	8C	8C	7Pm	AVI
		PCV	8BM	a10p	IFJa	IFJp
			PGs	a9-46v	8Av	
			IP2	i6-8	RSC	
					TE1m	
Limbic			EC	25		
			Amygdala	Amygdala		
			H	H		
DMN	47m	47m	47l	47l	31pd	31pd
	TPOJ2	POS1	POS1	55b	9p	
	STGa		8BL	v23ab	8Ad	
	STV		9m	STSvp	STSva	
			PGi	PSL	TE1a	
			s32	d32	TPOJ1	
DAN	LIPd	LIPv	PEF	PEF	LIPv	
		TE2p	6a			
		MST	AIP			
Salience	FOP1	FOP4	PFop	MI	FOP4	9-46d
	9-46d		SCEF		PFcm	
	23c				p32pr	
	6r				46	
					a24pr	
					PSL	
Visual	V1	PIT	FFC	V3B	VMV1	VMV1
	V3		V8	V8		V4
	V3B		LO2			
	IP0		PGp			
			ProS			
			TPOJ3			
						
Sensorimotor				LBelt	MBelt	2
				3a		52

Duplicate parcellations that appeared in both “ADHD” and “Controls” are listed in the third major column, “ADHD and controls”. Glasser’s parcellations matched with activated brain foci in ‘ADHD activation’ and ‘Controls activation’ groups.

Table 3 presents the matched Glasser’s parcellations and the affiliated brain networks of each activated brain foci in the “ADHD < controls” and “ADHD > controls” groups. More parcellations from the DMN limbic networks were activated to a greater extent in controls relative to children with ADHD. One parcellation from the DMN, area 47l, demonstrated unique hemispheric asymmetry, being activated on the left for ADHD and on the right for controls. Left-sided parcellations of the DAN such as 7 am, 7PL, and PH were more activated in ADHD, while right-sided parcellations such as PEF and 6a were more activated in controls. Both left and right p9-46v parcellation of the CEN network was more activated in ADHD along with other CEN parcellations such as PCV, IFJp, 8C, 44, and PFm.

**Table 3:** List of parcellations and matched networks activated in “ADHD > Controls” and “ADHD < Controls”

	ADHD > controls		ADHD < controls		ADHD = controls	
	R	L	R	L	R	L
CEN	p9-46v	p9-46v	IFSp	33pr	p24	
	PCV	p10p	RSC	RSC	p10p	
	IFJp	a47r		IFJa		
	8C			POS2		
	44					
	PFm					
Limbic	Hippocampus		Amygdala	25		
				H		
DMN	STSdp		STV	STSdp		
	31pv	45	47l	STSvp		
	8Ad	47l	47m	47m		
	9a	d23ab	7m	47s		
	TGd		9p	9p		
			a24	55b		POS1
			A5	9m		
			PHA3	d32		
			TPOJ1	PSL		
			TE1a	STGa		
DAN	LIPv	7Am	PEF	PFt		
		7PL	6a			
		PH				
Salience	9-46d		SCEF	MI	p32pr	
	PoI2		FOP4			
	46					
Visual	V1	V3B	VMV1	V1		
	V4	V4	VMV3	VMV3		
	V6		VVC			
	PH		FFC			
			DVT			
Sensorimotor	5m	OP4	2			4
		RI	3a	3a		

Duplicate parcellations that appeared in both groups are listed as “ADHD = Controls”. Glasser’s parcellations matched with activated brain foci in “ADHD > Controls” and “ADHD > Controls” groups.

Venn diagrammatic representation of parcellations activated in the “controls only” and “ADHD < controls” groups are presented in Figure 5 for the DMN, DAN, and the salience network. “Concordant” parcellation, that is parcellations belonging to both groups were areas 55b, d32, PSL, and STSvp on the left and areas 47l on the right for the DMN, areas PEF and 6a on the right for DAN, and areas MI (L) and SCEF (R) for the salience network. Figure 6 provides a summary of the networks activated predominantly by ADHD and control subjects as well as the distinct proportions of each network that were shared among both groups.

## 5 Discussion

A coordinate-based meta-analytical approach was employed to understand neural correlates of motor inhibition in children with ADHD during the Go/No-Go task, which was useful in summarising anatomical information pertaining to inhibitory effort or lack thereof in an unbiased and objective fashion. Furthermore, ALE generation combined with brain network-based nomenclature offers a greater degree of objective and quantitative specificity, allowing for investigation of regions of interest beyond macro-systems and lobes and into finer details of network affiliations and connectivity. We confirmed existing consensus stating broad involvement of the SMA and fronto-striatal circuits while providing additional insights regarding greater DMN and limbic network connectivity in controls, and unique hemispheric asymmetry of the DAN between controls and ADHD children. Additionally, considering the significant impact of ADHD on childhood development (affecting academic performance, interpersonal and family relations, conduct problems, substance experimentations, and abuse [36]) and the inherent differences between adolescents and adults in terms of prevalence [37], presentation [38], neurobiology [13,15], and impact of ADHD [36], we believe our meta-analysis offers clinical interest for the study of the pediatric ADHD population.

Our findings coincide with theories posited by other recent meta-analyses in neuroimaging literature for ADHD. First, control subjects activated more parcellations of the DMN than their ADHD counterparts. This suggests decreased DMN regulation and stability, despite DMN hyper-connectivity in ADHD [39], which could result in deficits like inappropriately “switching on/off” the DMN to fulfil response inhibition. The DMN – the “resting state” network, is a collection of neural nodes responsible for task-irrelevant mental processes, mind-wandering, and self-generated thoughts [40,41,42]. Some studies have suggested that an absence of inverse correlations between cognitive control networks (i.e. CEN) and the DMN reflects DMN intrusion during active tasks, manifesting as attentional lapses or inability to complete tasks [43,44,45,46,47]. Other hypotheses suggest decreased synchrony within the DMN, although findings across multiple studies have not been unanimous [16]. While many of these insights regarding aberrant DMN connectivity stem from the resting-state fMRI literature [9,4,16], the present fMRI meta-analysis considers the prevalence of significant task-based DMN activation differences.

We found that DMN hemispheric asymmetry, particularly for area 47l, is reproducible and valid. Several studies have suggested that the DMN demonstrates functional asymmetries, with the right-sided network composing the right inferior parietal lobule (IPL) responsible for simultaneously maintaining attention on current task goals and responding to salient information or environmental stimuli. However, the left IPL is more involved in language networks [48,49] – area 47l is known for its association with Broca’s area, contributions to the frontal-subcortical circuit and involvement in language functions and semantic processing [50,51]. Similar conclusions can be made regarding area 47s, another parcellation matched to a significant cluster in our analysis [51]. While these language-oriented functions may be applicable for left-hemispheric areas 47l and 47s, there is a lack of data explaining the function of these areas on the right in a right-dominant brain. Interestingly, “concordant” parcellations activated for controls in the DMN such as areas 55b, d32, PSL, and STSvp are also involved in language processing, and controls demonstrated more extensive connectivity in brain language centres during the task. Several studies have reported language deficiencies in ADHD subjects but none have presented evidence for language deficits during the Go/No-Go task [52,53]. We do not believe that there is a causal relationship between language-based learning difficulties and the lateralization of our findings for the DMN. However, we do highlight a co-occurrence of these factors. We also highlight a significant gap in the literature regarding the function of the aforementioned areas (47l, 47s) on the right side and the implications of DMN asymmetry. Overall, further studies to elucidate the nature and extent of DMN abnormalities are necessary.

Shifting focus onto complex network models of ADHD neural circuitry has revealed inappropriate engagement of attentional systems such as the salience network and the DAN [16,17]. We found unique hemispheric asymmetry in DAN activation, with ADHD and controls activating left- and right-sided parcellations of the DAN, respectively. The DAN, primarily composed of the intraparietal sulcus and frontal eye fields of both hemispheres, is active during spatial attention, feature-based attention, saccade planning, and visual working memory [54,55,56]. A significant “concordant” parcellation activated in controls but not in ADHD was the right PEF (parietal eye field) and area 6a. Area 6a along with 6d form subdivisions of the premotor cortex and while their exact functions are still unknown, the role of the premotor cortex in responding to visual or auditory cues to coordinate or plan voluntary movement is well established [57]. Corbetta and Shulman explained how DAN and ventral attention network interactions can lead to a unique dichotomy of attentional coordination. The former applies cognitive “top-down” selection for stimuli and its appropriate response, while the latter detects salient stimuli to act as a “circuit breaker” [58]. Applying this to our Go/No-Go paradigm, the DAN is responsible for attentional processes driven top-down, that is cognitive information like colours, shapes, or numbers (perceptual set) requiring actions like a button press (motor set), forming an “attentional set.” A second attentional network, heavily lateralized to the right brain including the right temporoparietal junction and right ventral frontal cortex, is then responsible to look out for salient and other potentially important stimuli (STOP signals) and interrupt or inhibit ongoing cognitive activity and the aforementioned attentional process. This may explain why ADHD subjects in this meta-analysis lacked right-sided DAN parcellation activation – that is they lacked the core inhibitor or “circuit-breaker” of goal-directed attentional processes lateralized to the right brain. It is known that patients with right-sided attentional network injury and dysfunction present with the clinical syndrome of neglect. Applying this to meta-analysis, the inability to inhibit motor responses during cognitive tasks requiring sustained attention may be a form of “neglecting” salient cues, arising from poor connectivity in right-sided attentional networks. This poses interesting questions: does the inability to inhibit motor responses in the ADHD phenotype stem from a mechanism distinct from deficits in sustained attention? Or, are the two core deficits intimately intertwined in their mechanistic processes? Future research separating the study groups based on these core deficits may shed further light regarding the directional relationship between these key aspects of ADHD.

Another finding was the comparatively minimal limbic network activation in ADHD children compared to controls. In the present-day literature, the core phenotype of ADHD has extended beyond just deficits in motor inhibition and sustained attention to include a variety of non-cognitive symptoms such as dissociated emotional regulation [59], hyper-aggressiveness [60], emotional lability [61], and depressive symptoms [62]. Some studies have attributed these affective symptoms of ADHD to altered limbic circuitry and amygdala activation [17,40]. For example, Posner et al. reported hypoconnectivity within regions of the limbic cortico–striato–thalamo–cortical loops to the presentation of emotional lability in people with ADHD [59]. However, the association between limbic network connectivity and performance in the Go/No-Go task remains unclear.

There are several notable considerations regarding our study. First, our meta-analytical method has yielded insignificant ALE clusters for the ADHD group at our current thresholding level. This is not unlike other meta-analyses which have also failed to find conclusive findings across several ADHD fMRI datasets [9,16,63]. There are several potential reasons for the abundance of negative findings in the ADHD fMRI literature: (1) slight differences in experimental paradigms and contrasts in task-based fMRI studies; (2) heterogeneity in the sample population in terms of age, sex, medication status, and severity of symptoms; (3) heterogeneity in the ADHD phenotype and genotype itself; and (4) random and systematic errors in the fMRI work-flow, e.g. head movements during task-based fMRI [17]. We posit that the significant convergence towards our ALE clusters for the control groups can be attributed to our inclusion of only the adolescent population, hence reducing heterogenicity.

On this note, heterogeneity and the quality of the included studies are key challenges faced by any meta-analyses. In response to this challenge, our study utilized ALEs. ALE assesses the convergence of activation coordinates across included studies, allowing research studies to identify regions of interest consistently associated with the Go-No/Go paradigm in children with ADHD. By aggregating findings from multiple studies, ALE provides a robust statistical framework that accounts for both the variability and consistency of reported activations, and thus aims to derive objective outcomes amidst a collection of heterogeneous data. We believe that this serves to overcome the limitations of individual studies and thus arrive at a more comprehensive understanding of the neurological correlates of motor inhibition in children with ADHD.

Despite the choice of ALEs being appropriate to address the research question, it is often difficult to balance between selecting enough studies to optimise analytic power and ensuring stringent inclusion criterion to minimise the confounding effects of heterogeneity. We estimate that while our meta-analysis had lesser heterogeneity, we lacked the statistical power to adequately analyse the ADHD population and may have allowed for a higher level of confounding. Furthermore, our study utilised Yeo et al.’s 7-network model of the brain with the intention to provide a foundational understanding of core neural correlates [19]. However, brain mapping is becoming increasingly nuanced in congruence with human connectomic complexity, and hence, future studies should use more detailed brain atlases such as Yeo et al.’s 17-network model [19]. Finally, while important, response inhibition is no longer the defining characteristic of ADHD and is now understood to be one of many different manifestations of complex neural circuitry. We recommend future studies exploring connectivity during other cognitively demanding tasks.

We were also limited by our database relying solely on the PubMed and BrainMap databases. PubMed is a widely recognized and frequently used database, especially in the field of neuroimaging studies. However, we believe that considerations for inclusion of other lesser-known databases in future research endeavours may yield a more comprehensive and holistic result. Finally, while our study focused primarily on the No-Go > Go contrast, future studies could explore the Go and No-Go activations separately in ADHD and control groups.

## 6 Conclusion

The present study summarises task-based fMRI data regarding the neural correlate of motor/response inhibition of children with ADHD during the Go/No-Go tasks using a coordinate-based meta-analytical method. ALEs were generated and matched to parcellations derived from the Human Connectome Project to understand brain connectivity at a network-based level. Our findings align with other recent meta-analyses conducted in this area. Of particular interest to this study was the less extensive activation of parcellations of DMN and the right-sided DAN in ADHD children.

## Supplementary Material

Supplementary material
