# Supplementary material for: Functional connectivity in ADHD children doing Go/No-Go tasks: An fMRI systematic review and meta-analysis
Source: Transl Neurosci. 2023 Dec 31;14(1):20220299. doi: 10.1515/tnsci-2022-0299 (PMC10896184; doi:10.1515/tnsci-2022-0299)
Supplement: Supplementary material [file tnsci-2022-0299-sm.pdf]

Supplementary material

Table S1: Summary of all 14 studies with MNI coordinates use to generate ALE

Study	Task	No. of subjects		Coordinates					
		ADHD	Controls	ADHD			Controls		
Booth et al., 2005	Subjects provided with one of three stimuli: red triangle (target stimuli), red trapezoid (distractor) and blue triangle (distractor). Subjects were instructed to button press with their fingers when presented with target stimuli and withhold otherwise	12	12	42	9	15	3	18	54
				−24	−51	51	15	57	21
				30	−60	3	27	0	42
				21	−60	48	42	24	15
				−3	−15	−21	−30	27	−12
				−33	30	6	48	−3	27
				−39	−48	3	−42	0	33
				48	−9	−24	−12	−3	18
							−42	−30	0
							−24	−78	12
							−30	−24	−9
							30	−54	57
							33	−33	36
							−30	−45	57
							45	−12	−18
							−21	−3	−18
				ADHD > Controls			ADHD < Controls		
							0	15	54
							24	−9	48
							45	3	45
							−21	30	−12
							42	27	15
							21	51	9
							9	42	6
							45	−6	24
							−36	9	21
							6	12	3
							12	−9	18
							−12	−3	18
							−9	−33	−6
							30	−3	−15
							−3	−72	3
							27	−63	−21
							3	−24	3
				ADHD			Controls		

(Continued)

Table S1: *Continued*

Study	Task	No. of subjects		Coordinates					
		ADHD	Controls	ADHD			Controls		
Durston et al., 2003	Standard Go/No-Go paradigm using popular children cartoon characters as target stimuli to which subjects were instructed to respond via button-press.	7	7	21	−46	−6	21	−46	−6
				−21	−47	−5	−21	−47	−5
				54	−34	26	54	−34	26
				8	−52	37	8	−52	37
				−9	−49	37	−9	−49	37
				18	52	24	62	−27	27
				19	−37	32	33	33	41
				−17	−46	37	9	29	−8
				48	−47	25	−44	22	41
				18	−84	−7	−16	6	16
				−13	84	8	26	−4	−12
				12	−43	49	−3	3	26
				−12	−31	45			
				ADHD > Controls			ADHD < Controls		
				12	59	24	−10	29	−8
				−20	86	4			
				15	90	−2			
				28	−44	24			
				5	−57	53			
				−4	−59	53			
				39	−35	0			
				42	30	25			
				19	−45	29			
				−8	−51	26			
				55	17	24			
				ADHD			Controls		
Durston et al., 2006	Standard Go/No-Go paradigm using popular children cartoon characters as target stimuli to which subjects were instructed to respond via button-press.	11	11	49	16	45	42	24	−14
				43	−44	50	−37	23	−13
							5	43	18
							−8	42	27
							−37	45	13
							45	12	37
							−45	4	56
							29	57	9
							−61	−51	29
				ADHD > Controls			ADHD < Controls		
						−37	23	−13	
						−8	42	27	
						−45	4	56	

(Continued)

Table S1: *Continued*

Study	Task	No. of subjects		Coordinates					
		ADHD	Controls	ADHD			Controls		
						29	57	9	
						−61	−51	29	
				ADHD > Controls			ADHD < Controls		
Hart et al., 2014	Subjects respond to right/left arrows with right/left thumb button press. Withheld responses for stop signs.	30	30	−26	−67	−13	29	30	−18
							−37	30	−14
							−3	18	−24
							−46	−40	2
							38	−58	−21
							−51	−46	26
							−7	−79	43
							21	−62	26
				ADHD			Controls		
Janssen et al., 2015	Subjects responded to pictures of airplanes with a button press and withheld their responses if pictures were superimposed with a white cross.	21	17	33	39	31	50	19	−1
				−32	19	4	−35	10	3
				5	18	36	6	36	16
							21	56	30
				ADHD > Controls			ADHD < Controls		
Ma et al., 2012	Subjects instructed to button press for any letter of the alphabet except for the letter 'V'.	15	15	44	−64	−8			
				4	−12	−14			
				34	0	28			
				32	−60	12			
				32	−74	−2			
				−32	−76	12			
				4	−40	−22			
				2	−80	−30			
				−54	−4	16			
				−48	−34	18			
				32	−28	−8			
				28	−90	14			
				−40	−84	−12			
				ADHD			Controls		
Pliszjka et al., 2006	Subjects were instructed to left thumb press for "A" and right thumb press for "B". Subjects asked to with-hold a response for "S"	16	15	41	15	7	41	5	29
				48	−54	11	55	−46	11
				24	−86	19	2	24	32
				−41	−74	−3	40	21	0
				30	−54	44	−44	24	−4
				44	4	28	37	−45	40
				−36	−3	37	−43	4	39
				39	15	10	43	7	37

(Continued)

Table S1: *Continued*

Study	Task	No. of subjects		Coordinates					
		ADHD	Controls	ADHD			Controls		
				−35	21	−3			
				3	20	34			
				−44	−74	−6			
				−29	−55	40			
				ADHD			Controls		
Rubia et al., 2005	Subjects respond to right/left arrows with right/left thumb button press. Withheld responses for up arrows	16	21	17	25	36	40	20	−13
				50	−4	1	−42	15	−7
				−41	−64	16	−27	59	−9
				−13	−38	49	35	52	5
							−22	13	43
							38	12	40
							43	0	9
							47	−51	35
							−27	−32	53
							36	−52	30
							8	15	0
							−4	−20	−23
							0	50	30
							54	−25	−16
							−58	−22	−11
							47	−14	0
							2	−58	19
				ADHD > Controls			ADHD < Controls		
							42	23	−13
							42	23	−20
							62	3	−8
							4	−58	24
							4	−66	26
							4	−42	13
				ADHD			Controls		
Schulz et al., 2004	Subjects were instructed to button press for letters between “A” to “F” and to withhold a response for the letter “X”.	10	9	46	26	−23	47	10	7
				−35	29	−21	−54	−14	8
				31	52	16	31	−73	−15
				37	19	27	33	−50	−24
				31	−68	30	−30	−50	−27
				−15	−64	25			
				ADHD > Controls			ADHD < Controls		
				29	54	15	−39	−18	38
				−22	64	6	50	2	−36

(Continued)

Table S1: *Continued*

Study	Task	No. of subjects		Coordinates					
		ADHD	Controls	ADHD			Controls		
				37	16	30	−36	−16	−23
				36	28	−25	16	−49	7
				−32	30	−25	4	−53	−22
				−6	−50	−8	−19	−61	−17
				−1	36	24			
				50	−44	33			
				−35	−33	28			
				16	−68	24			
				ADHD			Controls		
Spinelli et al., 2011	Subjects were instructed to button press when presented with a green spaceship and to withhold a response for a red spaceship	13	17	−14	−72	−26	42	−82	28
				−28	−66	−38	44	−70	34
				−16	−48	−26	44	−62	26
				20	46	28	20	−6	−28
				20	44	18	36	−16	−22
				−22	6	28	28	−10	−24
				−24	14	18	22	30	36
				−26	−4	8	22	24	42
				−12	18	10	14	30	48
				−12	12	18	−2	−62	22
				−12	0	24	18	−56	6
				ADHD > Controls			ADHD < Controls		
				22	48	28			
				20	44	20			
				24	50	2			
				−18	−64	−30			
				−18	−54	−28			
				−22	−58	−38			
				−50	30	−2			
				−46	36	−14			
				−54	36	−10			
				ADHD			Controls		
Suskauer, Simmonds, Fotedar et al., 2008	Subjects were instructed to button press when presented with a green spaceship and to withhold a response for a red spaceship	25	25	6	10	49	−37	−73	−13
							2	16	47
							45	−79	−4
							39	44	25
							49	−39	13
							6	−62	−17
							25	14	−7
				ADHD > Controls			ADHD < Controls		

(Continued)

Table S1: *Continued*

Study	Task	No. of subjects		Coordinates					
		ADHD	Controls	ADHD			Controls		
				6	−37	76	61	−37	13
							−11	−55	−41
							−27	−60	−12
							−24	−25	62
							30	−62	−12
							−1	11	56
							−24	−35	−32
							10	16	43
							52	−39	16
							−5	−40	19
							−32	−22	56
							−25	0	17
							−17	−51	20
				ADHD			Controls		
Tamm et al., 2004	Subjects were asked to keypress for every letter except "A" which occurred in 17% of trials	10	12	−54	−29	−19	41	−71	53
				70	−24	−13	5	40	42
				−42	16	13	46	24	−14
				34	31	−13			
				−4	48	12			
				ADHD > Controls			ADHD < Controls		
				−56	−33	−19	10	6	43
				ADHD			Controls		
Vaidya et al., 2005	Subjects were asked to left/right button press based of left/right direction of central arrow with flanking stimuli. Subjects were asked to withhold a response for the letter "X"	10	10	32	20	−7	35	−5	33
				55	−47	7	46	10	28
							−6	25	−5
							7	15	−8
				ADHD > Controls			ADHD < Controls		
Van Rooij et al., 2015	Subjects were asked to press right and left buttons corresponding to the direction of a picture of an aircraft, and to withhold their button press if there was a visual stop sign superimposed on the picture of the aircraft	185	235				−38	20	−18
							−2	60	38
				ADHD			Controls		
Wang et al., 2013	Subjects were asked to button press when the number "1" was followed by the number "9". Subjects were asked to withhold a response if "1" was followed by a number other than "9".	28	31	6	23	47	−13	19	34
				41	30	−9	0	−56	36
				−47	11	28			
				−27	−56	42			
				23	−50	44			
				6	−79	−24			

(Continued)

Table S1: *Continued*

Study	Task	No. of subjects		Coordinates					
		ADHD	Controls	ADHD			Controls		
				−47	10	31			
				−60	−32	−2			
				−42	−66	45			
				47	−60	45			
				−7	−53	44			
				ADHD > Controls			ADHD < Controls		
				−52	22	30	−4	8	26
				−44	−62	−2	33	−12	38
				−45	−80	−13	−56	−25	−10
				−22	12	−4	−31	−7	−22
				−10	−47	8	31	−26	−21
				−12	−71	56	25	−4	−22
				12	−53	−12			
				−19	22	22			
				−44	−59	2			
				20	−47	48			
				−28	−26	30			
				17	−78	−24			
